# Study of the Bacterial, Fungal, and Archaeal Communities Structures near the Bulgarian Antarctic Research Base “St. Kliment Ohridski” on Livingston Island, Antarctica

**DOI:** 10.3390/life14020278

**Published:** 2024-02-19

**Authors:** Vesselin V. Doytchinov, Slavil Peykov, Svetoslav G. Dimov

**Affiliations:** Department of Genetics, Faculty of Biology, Sofia University “St. Kliment Ohridski”, 1164 Sofia, Bulgaria; v.doichinov@uni-sofia.bg (V.V.D.); spejkov@biofac.uni-sofia.bg (S.P.)

**Keywords:** amplicon-based metagenomics, polar microorganisms, environmental microbiomes

## Abstract

As belonging to one of the most isolated continents on our planet, the microbial composition of different environments in Antarctica could hold a plethora of undiscovered species with the potential for biotechnological applications. This manuscript delineates our discoveries after an expedition to the Bulgarian Antarctic Base “St. Kliment Ohridski” situated on Livingston Island, Antarctica. Amplicon-based metagenomics targeting the 16S rRNA genes and ITS2 region were employed to assess the metagenomes of the bacterial, fungal, and archaeal communities across diverse sites within and proximal to the research station. The predominant bacterial assemblages identified included *Oxyphotobacteria*, *Bacteroidia*, *Gammaprotobacteria*, and *Alphaprotobacteria*. A substantial proportion of cyanobacteria reads were attributed to a singular uncultured taxon within the family *Leptolyngbyaceae*. The bacterial profile of a lagoon near the base exhibited indications of penguin activity, characterized by a higher abundance of *Clostridia*, similar to lithotelm samples from Hannah Pt. Although most fungal reads in the samples could not be identified at the species level, noteworthy genera, namely *Betamyces* and *Tetracladium*, were identified. Archaeal abundance was negligible, with prevalent groups including *Woesearchaeales*, *Nitrosarchaeum*, *Candidatus Nitrosopumilus*, and Marine Group II.

## 1. Introduction

With no permanent human habitation, the Antarctic continent represents one of Earth’s most isolated and remote places. An ample reason for this is the severe and unpredictable weather conditions, characterized by temperatures that may plummet below −60 °C in the interior regions of Antarctica during winter, where approximately 99% of the landmass is shrouded in snow or ice [1]. Recurring freeze–thaw cycles, intense UV radiation, scarce liquid water and substrates, and the lack of vegetation pose the most significant challenges for survival in this environment. However, despite the obstacles that face life in Antarctica, several places within the continent have been implicated as “biodiversity hotspots” and potential reservoirs of undiscovered species [2]. That is why Antarctica is far from a barren wasteland devoid of microbial life, as was the general notion before the mid-1980s, right at the cusp of a shift towards using molecular techniques for species annotation [3]. The most hospitable parts of the continent are found at the lower latitudes, especially in the Antarctic Peninsula and Maritime Antarctica.

Studying Antarctic microorganisms holds promise in multiple areas of research because of the unique environment they are found in. These organisms are well-adapted to low temperatures, intense UV radiation, and low nutrient input. As a result, some of their secondary metabolites and enzymes exhibit considerable potential for industrial applications [4,5]. Biotechnology often relies on enzymes capable of performing their catalytic functions at high or low temperatures. Exploring new species inherently bears the prospect of discovering novel antimicrobial compounds; some of the findings in this area have recently been reviewed [6]. Understanding how microorganisms adapt and how microbial profiles shift in response to rapid environmental changes is crucial for understanding how similar communities react to such perturbations. Some Antarctic studies that track the change in microbial profiles due to the influx of organic carbon sources [7,8] or temperature variations [9] have already been carried out. Such knowledge will allow us to preserve the Antarctic and other similar desert ecosystems better. Additionally, Antarctica’s pristine and isolated environments provide an excellent opportunity to study the effects of anthropogenic factors on microbial communities [10]. Research stations in Antarctica are recognized sources of anthropogenic influence on the continent, attributed to factors such as the deposition of microplastics from equipment [11] and an increase in antimicrobial-resistant bacteria [12,13].

The Bulgarian Antarctic Base “St. Kliment Ohridski” is a research station located in Maritime Antarctica at 62°64′ S, 60°36′ W, on Livingston Island, which is part of the South Shetland Islands archipelago. It was founded in 1988, but the microbial composition of the diverse ecological niches around the site remains unknown. These environments encompass a lagoon, multiple meltwater ponds and streams, the Todorina Buza tarn, and a recently formed unnamed lake. In 2022, an expedition to the station was carried out to collect samples for direct NGS metagenomic sequencing to assess the microbial composition of various sites around the base and identify noteworthy microorganisms.

## 2. Materials and Methods

### 2.1. Sampling Sites

**Figure 1:**
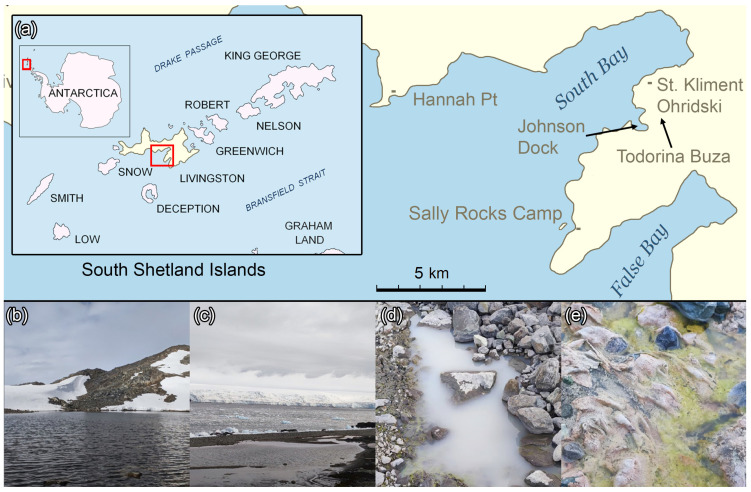
Location of the Bulgarian Antarctic Base “St. Kliment Ohridski” and some sampling locations. Description: (**a**)—location of the Antarctic base, (**b**)—Todorina Buza tarn, (**c**)—lagoon, (**d**)—one of the meltwater ponds, (**e**)—meltwater stream between the ponds.

Sampling was conducted as previously described [14] in January 2022 near the Bulgarian Antarctic Base “St. Kliment Ohridski” on Livingston Island, Maritime Antarctica (Figure 1).

The different samples with their descriptions and the corresponding environments are listed in Table 1.

### 2.2. DNA Extraction

Total DNA was isolated immediately after the collection to minimize potential shifts in the microbial compositions of the samples. DNA from the solid samples was extracted with ZR Soil Microbe DNA MiniPrep Kit (Zymo Research Corp., Irvine, CA, USA) according to the manufacturer’s Instructions manual. Two liters of water underwent filtration through a 0.2 µm filter for water samples. Subsequently, the biomass was rinsed off using 700 µL of the Bashing Beads buffer from the ZR Soil Microbe DNA MiniPrep Kit. The eluted biomass was then transferred to the kit’s Bashing Beads tubes and subjected to further processing in a manner identical to that employed for solid samples. The eluted DNA samples were quantified via fluorometric measurement (Quantus—Promega Corp., Madison, WI, USA) and kept at −20 °C.

### 2.3. Amplicon-Based Metagenomic Sequencing

Sequencing was carried out by the Novogene company. For bacteria, the V3-V4 16S rRNA regions were amplified using primers 341F (5′-CCTAYGGGRBGCASCAG-3′) and 806R (5′-GGACTACNNGGGTATCTAAT-3′) [15]. For archaea, the V4-V5 16S rRNA regions were amplified using primers Arch519F (5′-CAGCCGCCGCGGTAA-3′) and Arch915R (5′-GTGCTCCCCCGCCAATTCCT-3′) [16]. For fungi, the ITS2 region was amplified using primers ITS3-2024F (5′-GCATCGATGAAGAACGCAGC-3′) and ITS4-2409R (5′-TCCTCCGCTTATTGATATGC-3′) [17]. The corresponding libraries were sequenced on an Illumina platform, generating 2 × 250 bp paired-end reads.

### 2.4. Data Analysis

Sequence processing, data analysis, and visualization were carried out using the QIIME2 pipeline [18]. Barcode and primer removal, denoising, chimera removal, and pair-end joining were performed using the DADA2 plugin [19] to generate amplicon sequence variants (ASVs). Taxonomy classifiers were trained on the SILVA132 [20] and UNITE ver9.0 [21] databases after extracting the sequenced region through in silico PCR with the primers used in this study and using QIIME2’s feature-classifier plugin [22]. Taxonomy was assigned with a 0.8 confidence threshold, and chloroplasts were removed from the dataset [23]. Alpha diversity indices were calculated using the diversity plugin. For beta diversity analysis, a rooted phylogeny tree was constructed using the phylogeny plugin fasttree method and then used to calculate the weighted UniFrac distances and a PCoA matrix using the diversity plugins. Plots were made using the matplotlib and seaborn python packages [24,25].

## 3. Results

### 3.1. Sequencing Quality and ASV Counts

**Figure 2:**
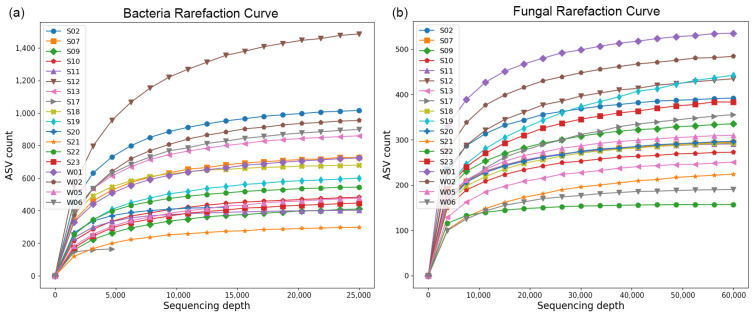
Rarefaction curvec. The samples’ richness (ASV counts) was plotted against the sequencing depths. (**a**)—bacterial; (**b**)—fungal.

A total of 5583 ASVs were obtained using eubacterial 16S primers, with only 4 ASVs common to all samples. A total of 2696 ASVs were obtained using fungal primers, with 5 of them being common to all samples. Most ASVs obtained using the archaea-specific primers were taxonomically identified as eubacterial. The eubacterial and fungal rarefaction curves are presented in Figure 2 and show that an adequate sequencing depth was achieved for both domains in all samples. The Q20 and Q30 scores before filtering and denoising were above 97% and 91%, respectively.

### 3.2. Taxonomic Annotation

**Figure 3:**
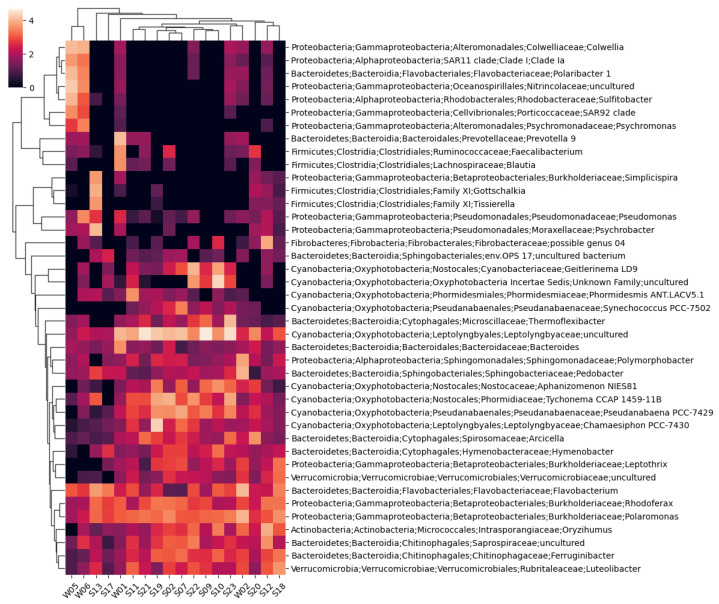
A cluster heatmap was constructed using Bray–Curtis dissimilarity between the bacterial taxa only for taxa representing at least 4% of sequences in any of the samples.

Taxonomic annotations representing at least 4% of eubacterial reads in any sample are shown on the cluster heatmap represented in Figure 3.

A total of 1894 unique bacterial taxons were identified, 1156 of which were annotated to the species level. Only two taxons could not be assigned to a phylum. The highest number of identified bacterial taxons and annotated species belonged to sample S12, while the lowest were in sample S17.

**Figure 4:**
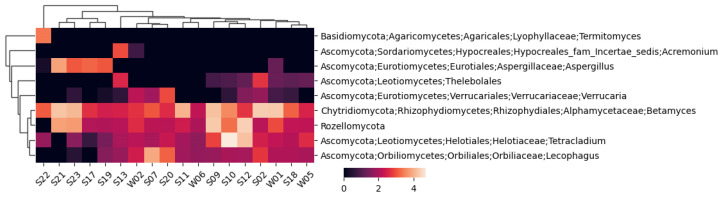
A cluster heatmap constructed using Bray–Curtis dissimilarity between the fungal taxa only for taxa representing at least 1% of sequences in any of the samples.

A similar fungal heatmap is given in Figure 4 but with a 1% cutoff.

For the fungi, 244 unique taxons were identified, 173 of which were annotated to the species level. The highest number of identified fungal taxons and annotated species belonged to sample S19, while the lowest were in sample W06.

**Figure 5:**
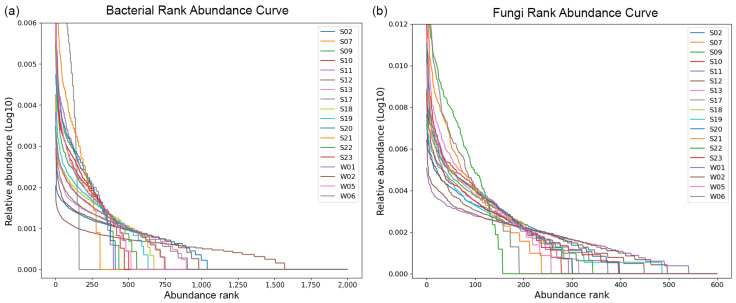
Rank abundance curves. The relative abundance (log_10_) of the (**a**) bacterial and (**b**) fungal ASVs in the samples plotted against their abundance rank. Steep curves show a community with low evenness, i.e., a few ASVs represent most sequences. The richness of a community can also be gauged by the abundance rank.

The evenness and richness of the bacterial and fungal communities are represented in the rank abundance curves in Figure 5a,b, respectively.

Sample W06 was isolated from the surface of a rock within the research base and displayed the lowest bacterial evenness and richness of any community. Only three organisms make up more than 50% of sequences in that sample. S12 is a soil sample underneath a vegetation patch and shows the highest richness of any bacterial community. Sample S09 displayed a very high evenness but low richness for the fungal community and is one of the microbial surface mat samples.

**Figure 6:**
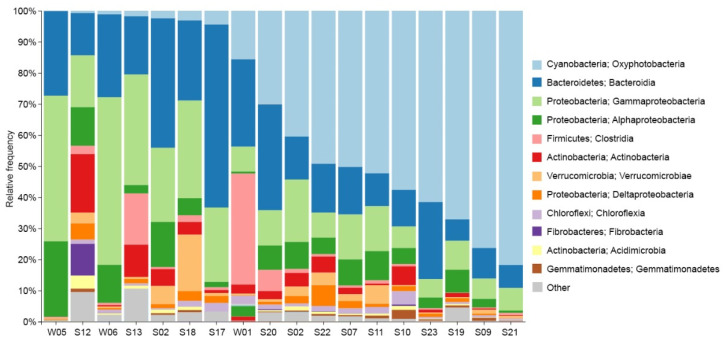
A bar chart showing the relative abundances of the dominant bacterial classes and the phylum they belong to.

The relative abundances of dominant bacterial classes for each sample, along with their respective phyla, are given in Figure 6. The most prominent group across most samples was a cyanobacteria belonging to *Leptolyngbyaceae*, constituting 79% of reads in one single sample. Moreover, cyanobacteria represented more than 50% of reads of all bacterial surface mats from submerged rocks. Other prevailing groups observed in many samples comprised *Bacteroidetes*, *Gammaproteobacteria*, and *Alphaproteobacteria*. *Bacteroidetes* represented 59% of reads in the dryland surface mat sample (S17). The water samples exhibited a high prevalence of *Proteobacteria*, up to 71% of the total sequencing reads with assigned taxonomy ranks. The class *Clostridiales* represented 36% of reads from the lagoon, a much higher value than in any other sample. Epilithic sample S17 from a rock inside the research base was characterized by a great abundance of *Flavobacterium* (37% of sequences), *Hymenobacter*, and, to some extent, *Rhodoferax*.

A total of 50–98% of fungal reads could not be assigned to a phylum. Sequencing using archaea-specific primers produced a small number of reads, representing <1% of sequences in most samples, while the rest were taxonomically identified as bacteria. The only exceptions were the seawater samples—W05 and W06, where archaea represented 41% and 8% of reads, respectively. Candidatus *Nitrosopumilus*, *Nitrosarchaeum,* and Marine Group II represented the majority of sequences from the seawater samples. S07 and S12 also had a slightly higher abundance of archaea, with 3% and 4%, respectively, and the archaea there were dominated by representatives of *Woesearchaeales*. The fungal genus *Betamyces* was prevalent in all samples isolated from the lagoon and the meltwater ponds, with an average of 13% of total reads.

### 3.3. Alpha Diversity Analysis

Alpha diversity metrics for bacteria are shown in Table 2.

Sample S12 has the highest bacterial richness and diversity of any sample, although the Simpson index is higher for some other samples. The lowest richness belongs to sample S17, but samples S21 and S23 have the lowest diversity and are both part of microbial surface contaminations.

The corresponding Alpha diversity metrics for fungi are given in Table 3.

Overall, fungal richness was much lower than bacterial. Freshwater samples W01 and W02 have a higher-than-average richness and diversity, yet phylogenetic diversity is even higher in some microbial surface mat samples. The lowest fungal richness and diversity by any metric were observed in saltwater sample W06.

### 3.4. Beta Diversity Analysis

**Figure 7:**
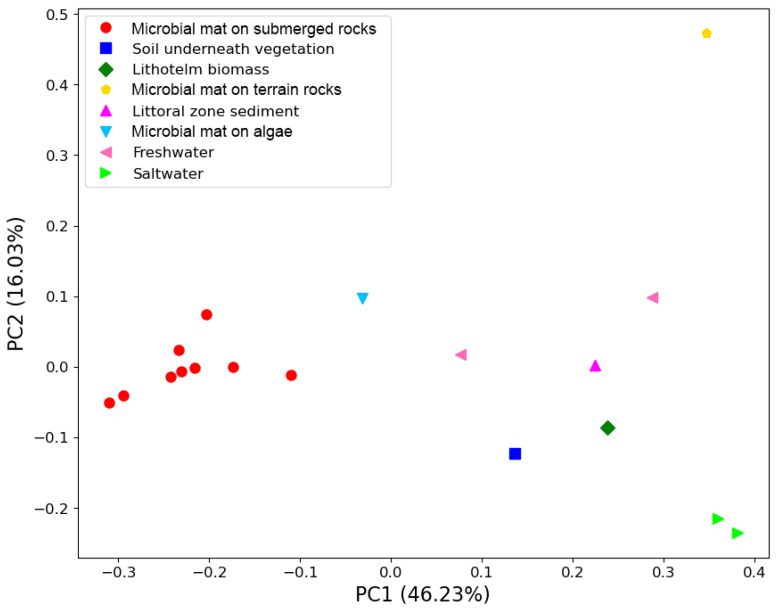
PCoA plot using weighted Unifrac distances between the samples for the bacterial reads.

The beta diversity of bacteria is represented using a PCoA plot and weighted UniFrac distances in Figure 7.

Based on the weighted UniFrac distance, all the microbial submerged rocks surface mat samples clustered together. The main driver for this clustering was the high abundance of photosynthetic cyanobacteria from the family *Oxyphotobacteria*. The most abundant ASVs could not be assigned beyond the family *Leptolyngbyaceae* and ranged from 19 to 78% of reads in these samples. Other genera of cyanobacteria that were common included *Tychonema* CCAP 1459-11B, *Pseudanabaena* PCC-7429, *Chamaesiphon* PCC-7430, *Geitlerinema* LD9, and *Aphanizomenon* NIES81. Sample S10 contained one ASV that could not be identified beyond *Oxyphotobacteria* yet represented 34% of reads. Sample S11 also contained a high abundance of the genus *Phormidesmis* ANT.LACV5.1.

Sample S12, taken from underneath a patch of vegetation near the nameless lake, boasts the highest alpha diversity of any sample and cannot easily cluster with any other communities based on beta diversity.

## 4. Discussion

### 4.1. General Sequencing Statistics Analyses and Observations

Rarefaction curves for bacterial and fungal sequences reach a plateau as the sequence depth increases, indicating that the samples for bacteria and fungi have attained adequate sampling depth. The observed species richness also strongly correlated with the richness estimators Chao1 and ACE. Overall, the samples exhibited substantial diversity concerning Simpson, Shannon, and phylogenetic diversity indices for bacteria and fungi, with minor exceptions noted in the cases of samples S09 and S21. Utilizing weighted UniFrac distances, the beta diversity analysis revealed a robust correlation between the bacterial communities and both the sample type and the sampling location, resulting in cohesive clustering based on various taxa. However, no comparable clustering was observed for fungi. The diminished richness and remarkably steep rank abundance gradient observed in sample S17 can be elucidated by the elevated count of chloroplasts, which constituted 94% of the obtained reads. A relatively modest number of bacterial ASVs were retrieved after excluding them from the dataset. Nevertheless, sample S17 reached a plateau on the rarefaction plot, and the observed richness aligned with the richness estimators, confirming its representativeness for the respective community.

Most fungal sequences in all samples could not be assigned to a given phylum. A high number of unidentified fungal species in Antarctic samples is not uncommon and highlights our limited understanding of this domain in both the Arctica and Antarctica [26]. Only a limited number of sequences accurately characterizing fungi from these regions are currently available within online databases, including UNITE, which was used to train the classifier in this study. A BLAST search was also performed, yielding an equally poor taxonomic identification of ASVs. Still, the unusually high share of unidentified ASVs around the Bulgarian Antarctic base “St. Kliment Ohridski” pointed to a hotspot of novel fungal species that requires further investigation. High amounts of unclassified fungal tags were reported also in another study on Livingston Island in particular [27], as well as in other studies in Maritime Antarctica in general, in some of which they have been reported to be predominant [28,29]. Investigating such locations can contribute significantly to understanding the fungal diversity in polar regions, offering potential biotechnological applications, including drug discovery. Additionally, the exploration is warranted for the potential presence of human pathogenic strains entombed in permafrost, as previously reported [30,31].

A relatively few archaeal reads were retrieved in the present study, with this domain being entirely absent in some samples. A limited number of taxa predominated the majority of archaea in the samples, indicating a low richness in the community. Although some archaeal species have been studied for possible biotechnological application [32], this domain represents another poorly understood group of organisms in Antarctica, probably because few species can be cultivated and studied in depth [33]. One of the approaches to overcome this problem is by extracting genomic data from metagenome samples or through metatranscriptomic approaches as has been reported [34].

### 4.2. Community Composition of Microbial Surface Mats on Submerged Rocks

The results show a high abundance and richness of cyanobacteria in these samples, all belonging to the family *Oxyphotobacteria*, which comprises a large group of oxygenic phototrophs [35]. That shows that bacterial surface mats on rocks in Antarctic meltwater ponds and meltwater streams near the research base contain a large number of cyanobacterial species that are yet to be identified. Such organisms could hold biotechnological potential to produce pigments, extracellular polysaccharides, antimicrobial compounds, and other valuable products [36,37,38].

Phyla other than cyanobacteria expressed very high richness and evenness at the genus level, except for sample S23, where the genus *Thermoflexibacter* accounted for 15% of all reads. In Antarctic freshwater bodies, bacteria commonly adopt a distinctive structure within biofilms and microbial mats. Cyanobacteria, particularly in the upper layers, play a dominant role as autotrophic producers for the community. In contrast, a diverse array of other bacteria in the lower layers assimilate the generated products [39,40,41,42].

Only about a quarter of fungal reads could be assigned to a phylum. *Betamyces* is a genus shown to dominate fungal communities formed on microplastics in a freshwater Arctic lake [43]. The presence of this genus could be interpreted as an indicator of increased microplastics in the water. Despite the fact that we do not have any proof that this is also the case in this research, such a hypothesis could not be completely excluded. A basis for such speculation is the fact that microplastics have also been implicated as a potential vector for the spread of antibiotic-resistant bacteria [44,45], as well as some published findings during the same Antarctic expedition reporting the presence of antibiotic-resistance genes in animals around the Bulgarian Antarctic base [12]. Surprisingly, sample S10 contained a very high abundance of a single genus—*Tetracladium*, which accounted for half of all reads and has been shown to have biotechnological potential for producing pectinolytic enzymes [46]. The phylum *Rozellomycota* was also identified, but no further taxonomic annotation was possible, and it was only detected in a significant abundance in sample S09.

Archaeal abundance in the submerged rock surface mat samples was primarily below 0.1% of the reads, except for S07, where they constituted 3% of all reads. In all samples, the genus *Woesearchaeales* from phylum *Nanoarchaeota* dominated. This genus has been shown to be prevalent in glacial meltwater, and its higher abundance here may be from a similar source [47]. *Woesearchaeales* have also been shown to be symbiotic with methanogenic archaea, and some members of the genus have been implicated in methane production [48,49]. It has been reported that halophile Archaea could be sources of industrially important biomolecules like, for example, proteases, amylases, surfactants, antifreeze, and antidesiccation proteins [50]. They also have a great potential for low-temperature biogenic methane production [51] or even medical use, for example, by producing radioprotective secondary metabolites [52].

### 4.3. Community Composition of a Microbial Surface Mat from an Epilithic Sample within the Research Base

Several *Flavobacterium* species have been isolated from polar soils, and multiple ASVs from this study were assigned to this genus [53]. The predominant presence of *Flavobacterium* is the primary factor contributing to the substantial disparity in beta diversity observed in this sample. An excess of algae can alter the composition of the bacterial community towards a distinct profile [54]. This hypothesis is supported by the elevated prevalence of chloroplast reads in the sample. Epilithic communities can also have a relatively unique set of oligotrophic microorganisms [55]. *Hymenobacter* is a genus with *H. roseosalivarius* as a type species that was isolated initially from sandstone in the McMurdo Dry Valleys, Antarctica. Still, the genus itself contains species isolated from harsh environments worldwide [56]. Archaea and identifiable fungi were negligible in this sample.

### 4.4. Community Composition of the Littoral Zone Sediment from Tarn Todorina Buza

Sediment sample S18 from the shallow littoral zone of the Todorina Buza tarn unsurprisingly clustered closest in beta diversity to the water sample from the same tarn. The variation in sediment composition can be attributed to the significantly higher abundance of the genus *Luteolibacter* and the notably lower levels of *Pedobacter* and *Polymorphobacter*, along with, to some extent, *Flavobacterium* and *Polaromonas*. Overall, the sediment sample boasts a higher alpha diversity than the water column and could serve as a possible reservoir of microorganisms for the water of the tarn. *Flavobacterium* is more prevalent in the water column of shallower Antarctic lakes, correlating with its high presence in the sediment, and similar mixing could occur with the other genera [57]. Members of the genus *Luteolibacter* have been isolated from Arctic soils and Antarctic hypersaline brines, which could point to the more significant terrestrial influence at the littoral zone of the tarn [58,59]. The only identified fungal genus that represented at least 1% of reads was *Betamyces*, while the archaeal fraction of prokaryotes was negligible. It is important to note that fungi do not constitute a substantial fraction of the biomass in aquatic ecosystems. However, they exhibit high richness, particularly in the littoral zone, which partially accounts for the abundance of novel reads [60].

### 4.5. Community Composition of the Soil Sample underneath a Patch of Vegetation near the Nameless Lake

Most genera accounted for 1–2% of reads each, which reflects the sample’s high richness and evenness; only two genera represented more than 10%—*Oryzihumus* and a possible genus from the family *Fibrobacteraceae*. This sample exhibited the highest relative abundance of Actinobacteria among all samples in our study. Coupled with the elevated prevalence of Proteobacteria and Bacteroidetes, it mirrors a bacterial composition akin to previous Antarctic rhizoanalyses [61,62]. The high diversity could be attributed to the properties of the plant rhizosphere. Studies in more temperate climate zones have shown decreased bacterial diversity in the plant rhizosphere than in the surrounding bulk soil due to selective interactions between the plant and the bacterial species [63]. However, Antarctic plants have also been shown to function as harbors against the unfavorable conditions of cold, oligotrophic deserts by moisture retention, easing temperature stress, preventing desiccation, and providing nutrients to the community [62].

The rhizosphere sample also contained one of the highest shares of identifiable fungal phyla, accounting for half of all reads. These fungi were shown to belong to *Ascomycota* and *Rozellomycota*. The *Ascomycota* genus *Tetracladium* represented most of the phylum, similar to the bacterial surface contamination sample S10 from submerged rocks. *Tetracladium* species have been shown to be beneficial root endophytes in terrestrial environments and could thus play an essential role in the rhizosphere of Antarctic plants [64]. *Rozellomycota* is a predominantly unfamiliar fungal phylum lacking chitinous cells. They operate as parasites towards molds, algae, crustaceans, and amoebae. Such interactions may confer benefits to the host plant in the rhizosphere [65]. The connection between the higher abundance of identifiable fungal phyla and the presence of a plant rhizosphere, which creates a more hospitable microenvironment correlating to conditions closer to those of more temperate regions, has been proposed before [9]. These conditions could promote the development of more cosmopolitan species, which are more easily identified due to a higher abundance of known sequences available to the classifier.

Archaea in the soil sample exhibit a higher abundance than other sampling sites, constituting 4% of the total reads. Most of them again belonged to the genus *Woesearchaeales* within the phylum *Nanoarchaeota*. This genus was also prevalent in one of the bacterial surface contamination samples from submerged rocks—S07. Considering that S07 and S12 have very different community profiles, the great variety of environments *Woesearchaeales* have been isolated from, and their largely unknown metabolism, it is difficult to assess the source of this particular archaeon or its function in the community [66].

### 4.6. Community Composition of a Microbial Mat from the Surface of an Algae

Sample S20, representing the algae surface mat, clusters most closely in composition with the submerged rock surface samples, as anticipated due to their similar nature. This sample also harbors a substantial proportion of Oxyphotobacteria, predominantly represented by an ASV from the family *Leptolyngbyaceae.* The primary distinction between both communities lies in the elevated abundance of the genus *Arcicella*, constituting 26% of reads in sample S20 while being nearly absent from the rock surface samples. High abundances of this genus in a freshwater lake have been shown to correlate with algal blooms, and their increased presence on the surface of algae is not surprising [67]. *Arcicella* species have also been previously found on the rock–water interface of Antarctic freshwater ecosystems [68], although, in our study, the genus was confined to the surface of algae. Archaea were not detected in this sample.

Once again, only a few fungal reads could be assigned to a phylum level. *Ascomycota* accounted for most of them, reaching up to 3% of the total reads. The identified genera from the phylum include *Lecophagus*, *Verrucaria*, and *Tetracladium*. *Lecophagus* is a genus that includes a variety of predatory fungi, including *L. antarcticus*, which infects tardigrades [69].

### 4.7. Community Composition of Lithotelm Biomass from Hannah Pt

Lithotelms are crevices inside rocks that accumulate biomass primarily from the activity of penguins near Hannah Pt. but also contain a lot of trapped seawater. Their unique characteristics contributed to a distinct community profile that does not exhibit specific clustering in weighted UniFrac beta diversity with any other sample. Furthermore, this is one of the most diverse samples in this study, characterized by a substantial number of ASVs that could not be assigned beyond the family level. The most representative genera included *Psychrobacter*, *Gottschalkia*, *Flavobacterium*, *Simplicispira*, and *Tissierella*, which have all been previously identified within penguin guano using molecular techniques [70]. *Psychrobacter* includes halotolerant and psychrotolerant species commonly isolated from Antarctic marine environments [71,72]. Recent studies have demonstrated that certain Psychrobacter strains can produce antibiofilm and polyethylene biodegrading enzymes that remain active at low temperatures. Therefore, novel Antarctic species within this genus could harbor significant biotechnological potential [73,74]. Members of this genus have also been proposed as probiotic candidates, and they may be part of the active microbiome in penguin intestinal tracts [75]. Fungi could not be identified to the phylum level, and archaea were absent from the lithotelm sample.

### 4.8. Saltwater Community Composition

The relatively high abundance of the genus *Cowellia* in the sea samples was previously reported in a survey of the sea-surface microlayer (SML) of the waters in the same area [76]. The same study pointed to a high abundance of *Pseudoalteromonas* and *Pseudomonas*, which could be constrained to the SML. Therefore, we did not see such a high abundance of those genera. *Cowellia* is a genus of generalist marine psychrotrophs that have been shown to metabolize a wide range of hydrocarbon compounds and are found in great abundance at oil spills [77]. *Sulfitobacter*, an unclassified genus of *Nitrincolaceae*, *Polaribacter*, SAR11 Clade Ia, *Pseudomonas*, and *Psychromonas* were some of the other predominant genera in shaping the profile of this community. A total of 98–99% of the fungal reads could not be assigned to a phylum level.

The seawater samples had the greatest share of archaeal reads when using archaea-specific primers. The archaea from South Bay (W06) were dominated almost exclusively by the genera *Nitrosarchaeum* and *Candidatus Nitrosopumilus* from the phylum *Thaumarchaeota*. Still, Marine Group II archaea from Euryarchaeota were also present with about 5% of the reads. *Thaumarchaeota* (formerly Marine Group I) have been shown to be more abundant at greater depths than MGII archaea [78]. Despite this, they represented most of the archaea from the littoral zone sample taken from South Bay, which could be due to mixing from waves at the shoreline. The genus *Nitrosarchaeum* does not contain species isolated from marine environments; it only thrives in low-salinity aquatic environments, which could be due to mixing from the freshwater lagoon or the presence of an unknown member of the genus. Both *Nitrosarchaeum* and *Candidatus Nitrosopumilus* represent mesophilic, neurophilic, aerobic, autotrophic, ammonia-oxidizing archaea [79,80]. Members of *Candidatus Nitrosopumilus* are among the most abundant archaea in marine environments and play a key role in marine nitrification. MGII were far more prevalent in the Johnson Dock sample (W05), possibly due to being sampled from the pelagic zone. MGII are commonly found in shallow water, potentially playing an important role in carbon cycling [81].

### 4.9. Freshwater Community Composition

The bacterial communities of the two freshwater samples did not cluster together based on weighted UniFrac distances. In fact, the freshwater sample W01 from the lagoon clustered separately from all other samples on the third axis of the PCoA plot. Freshwater sample W01 from the lagoon was characterized primarily by the genera *Prevotella*, *Faecalibacterium*, *Bacteroides*, *Bifidobacterium,* and the family *Lachnospiraceae*, which were practically absent from any other sampling location. The *Bacteroides–Prevotella* group is a reliable fecal pollution indicator related to the activity of warm-blooded animals [82]. Furthermore, the genus *Faecalibacterium* currently encompasses only a single species, *F. prausnitzii*, which, interestingly, is recognized as a predominant bacterium in the human colon [83]. However, whether the identified ASV in this study represents the existing species within the genus or a novel one remains uncertain. Members of the family *Lachnospiraceae* and the genus *Bifidobacterium* are some of the most abundant microbes in the intestinal tract of humans and animals [84,85,86]. All these genera make up 55% of the total reads, pointing to significant contamination in the lagoon caused by gut microorganisms. This is most likely a direct result of the known penguin activity in the lagoon. Overall, W01 had the largest relative abundance of Firmicutes in all samples of the present study. The same ASV from the family *Leptolyngbyaceae* is once again present in a relatively high abundance. Archaea represented less than 1% of the total sequences. A total of 75% of fungal sequences from sample W01 could not be matched to a phylum, and 21% were classified as *Betamyces*.

The bacterial composition of freshwater sample W02 from the Todorina Buza tarn stood out due to the elevated prevalence of the genera *Pedobacter* and *Polymorphobacter*, along with a notable presence of *Polaromonas* and, to some extent, *Flavobacterium*. *Pedobacter* and *Polymorphobacter* are cosmopolitan genera found in various habitats, and both have been previously identified as part of Antarctic lake communities [87]. *Polaromonas* is a genus commonly isolated from polar glaciers and other high-altitude locations [88]. Notably, *Pedobacter* species have demonstrated significant biotechnological potential in producing various compounds, encompassing chitinase, antioxidants, pigments, antimicrobial agents, and more [89,90,91]. A total of 98% of fungal sequences could not be assigned to a phylum, while archaea represented less than 1% of 16S reads.

## 5. Conclusions

The current study aimed to enhance our understanding of the microorganisms in and around the Bulgarian Polar Base “St. Kliment Ohridski” by employing high-throughput amplicon sequencing to profile the community composition and identify microorganisms of potential interest. The results unveiled substantial potential within uncultured *Oxyphotobacteria*, particularly a member of the family *Leptolyngbyaceae*, prevalent in nearly all the microbial surface mat samples from submerged rocks within the meltwater ponds. The heightened prevalence of unidentified fungi, although a common characteristic in Antarctic metagenomic studies, was particularly pronounced in our results. That underscores the imperative for further research in this domain within polar regions. Interestingly, the genus *Tetracladium* was one of the identified ASVs and has been shown to have potential for biotechnological applications. Archaea represented a minimal amount of the overall microorganism diversity, with abundance constrained primarily to the sea samples. Overall, our work sheds light on the previously unknown microorganisms near the research base and points to some areas of interest for further studies. We revealed that the lagoon near the base showed clear signs of contamination from various bacteria due to penguin activity, but pathogens were not detected. Finally, the fungal genus *Betamyces* was identified in the lagoon and adjacent meltwater ponds. Species within this genus have been previously documented as opportunistically growing on microplastics. This observation serves as an indicator of potential pollution with microplastics in the studied environments.

## Figures and Tables

**Table 1:** Sampling sites.

Samples	Environment	Description	Coordinates
**Solid samples (S)**			
S02	Fresh water lagoon	Submerged rock, microbial mat	−62.641324, −60.368854
S09, S10, S11, S21, S23	Meltwater ponds	Submerged rocks, microbial mats	−62.641450, −60.356733
S07, S19	Sea Lion Tarn	Submerged rocks, microbial mats	−62.647727, −60.353677
S17	Meltwater current inside the base	Submerged rock, microbial mat	−62.641241, −60.361171
S20	Sea Lion Tarn	Macroalgae surface, microbial mat	−62.647727, −60.353677
S13	Lithotelm in Hannah Point	Sludge biomass	−62.653262, −60.607902
S22	Unnamed freshwater lake	Submerged rock, sludge biomass	−62.640819, −60.350725
S12	Patch of vegetation near the nameless lake	Soil	−62.640819, −60.350725
S18	Sea Lion Tarn	Sediment	−62.647727, −60.353677
**Water samples (W)**			
W01	Fresh water lagoon	Freshwater	−62.641324, −60.368854
W02	Sea Lion Tarn	Freshwater	−62.647727, −60.353677
W05	Pelagic zone of Johnson Dock	Marine water	−62.659572, −60.370434
W06	Littoral zone of South Bay	Marine water	−62.638681, −60.367835

**Table 2:** Alpha diversity indices of all samples in the study based on the eubacterial reads.

Sample ID	Richness Index	Shannon Index	Simpson Index	Chao1 Index	ACE Index	Good’s Coverage	Faith PD
S02	1065	7.470	0.969	1070.884	1071.221	1.000	74.103
S07	760	6.665	0.939	762.935	763.534	1.000	62.509
S09	445	3.035	0.543	446.375	448.276	1.000	49.169
S10	513	5.207	0.871	515.619	515.698	1.000	47.562
S11	405	5.910	0.929	405.875	407.058	1.000	47.661
S12	1592	8.487	0.985	1595.281	1596.486	1.000	89.834
S13	906	7.760	0.987	907.324	908.300	1.000	52.236
S17	169	5.784	0.954	169.667	170.608	0.999	28.950
S18	694	7.762	0.988	697.889	697.478	1.000	52.832
S19	646	5.880	0.948	647.774	648.614	1.000	46.071
S20	422	5.601	0.893	424.294	425.636	0.999	45.472
S21	307	2.180	0.390	307.036	307.468	1.000	39.399
S22	562	6.201	0.960	562.294	563.108	1.000	64.900
S23	487	4.010	0.776	491.138	491.869	1.000	49.020
W01	772	7.122	0.976	776.636	777.081	1.000	56.188
W02	1006	6.462	0.945	1009.288	1010.519	1.000	65.424
W05	531	5.460	0.942	533.528	535.345	1.000	41.971
W06	948	7.551	0.982	961.892	963.056	0.999	68.004

**Table 3:** Alpha diversity indices of all samples in the study based on the fungal reads.

Sample ID	Richness Index	Simpson Index	Shannon Index	Chao1 Index	ACE Index	Good’s Coverage	Faith PD
S02	397	5.219	0.912	397.000	397.269	1.000	59.609
S07	301	4.147	0.834	301.000	301.255	1.000	46.483
S09	343	5.214	0.935	343.000	343.000	1.000	54.016
S10	278	3.756	0.813	278.000	278.000	1.000	47.546
S11	293	4.616	0.899	293.000	293.000	1.000	49.715
S12	450	5.283	0.923	450.000	450.297	1.000	73.568
S13	257	3.688	0.842	257.000	257.000	1.000	47.903
S17	387	3.449	0.718	388.950	391.773	1.000	72.876
S18	300	3.912	0.857	300.000	300.000	1.000	50.124
S19	488	4.591	0.869	488.006	488.922	1.000	98.081
S20	302	3.844	0.825	302.000	302.255	1.000	49.148
S21	240	2.833	0.643	240.120	240.865	1.000	65.513
S22	157	3.607	0.733	157.000	157.000	1.000	30.662
S23	399	4.730	0.887	399.000	399.268	1.000	98.842
W01	545	6.623	0.971	545.118	546.274	1.000	79.676
W02	497	5.043	0.878	497.000	497.000	1.000	74.793
W05	314	4.546	0.899	314.000	314.000	1.000	59.378
W06	191	2.604	0.609	191.000	191.000	1.000	36.959

## Data Availability

The raw sequencing reads of this study have been deposited in the Nacional Center for Biotechnology Information of the National Library of Medicine as sequence reads archives under the following accession numbers: PRJNA979344 (for Archaea), PRJNA979782 (for Fungi) and PRJNA979776 (for Bacteria). All BioProjects were registered on 3 June 2023.
